# No-reflow phenomenon during percutaneous coronary intervention in a patient with polycythemia vera: A case report

**DOI:** 10.1097/MD.0000000000019288

**Published:** 2020-02-28

**Authors:** Yudi Her Oktaviono, Suryo Ardi Hutomo, Makhyan Jibril Al-Farabi

**Affiliations:** a Department of Cardiology and Vascular Medicine, Faculty of Medicine, Universitas Airlangga, Soetomo General Hospital, Surabaya, Indonesia; b School of Healthcare Managemenent, University College London, Bloomsbury, London, United Kingdom.

**Keywords:** balloon dilatation, blood cancer, ST-elevation myocardial infarction, thrombectomy, total occlusion

## Abstract

**Rationale:**

Acute myocardial infarction is the leading cause of mortality and morbidity in a patient with polycythemia vera (PV). However, the benefit of various percutaneous coronary intervention (PCI) technique on the patient with PV is relatively unexplored.

**Patient concern:**

A 46-year-old woman presented to the primary hospital complained about new-onset typical chest pain. Echocardiography examination showed inferior ST-elevation myocardial infarction (STEMIs) and increased cardiac markers. Complete blood count showed elevated hemoglobin, white blood cell, and platelet.

**Diagnosis:**

Coronary angiography revealed simultaneous total occlusion at proximal right coronary artery (RCA) and also at proximal left anterior descending (LAD) artery. Elevated hemoglobin and hematocrit with JAK2 mutation establish the diagnosis of PV.

**Interventions:**

We performed multi-vessel primary PCI by using direct stenting in RCA and aspiration thrombectomy in LAD after failed with balloon dilatation and direct stenting method. This procedure resulted in thrombolysis in myocardial infarction (TIMI)-3 flow in both coronary arteries. However, the no-reflow phenomenon occurred in the LAD, followed by ventricular fibrillation. After several attempts of resuscitation, thrombus aspiration, and low-dose intracoronary thrombolysis, the patient was returned to spontaneous circulation. The patient then received dual antiplatelet and cytoreductive therapy.

**Outcomes:**

The patient clinical condition and laboratory finding were improved, and the patient was discharged on the 7th day after PCI.

**Lessons:**

Cardiologist should be aware of the no-reflow phenomenon risk in the patient with PV and STEMI. Direct stenting, intracoronary thrombectomy, and thrombolysis are preferable instead of balloon dilatation for PCI technique in this patient.

## 1 Introduction

Polycythemia vera (PV) is currently classified as myeloproliferative neoplasms (MPN) by the World Health Organization (WHO).^[1]^ In the patient with classic myeloproliferative neoplasms, acute myocardial infarction is the leading cause of mortality and morbidity, which happened in 9.4% of PV patients.^[2]^ To restore coronary blood flow in the acute ST-elevation myocardial infarction (STEMI), primary percutaneous coronary intervention (PCI) is the preferred reperfusion strategy. Various technical recommendation of primary PCI has been established for various type of coexisting disease.^[3]^ While the pathophysiology of STEMI in classic myeloproliferative neoplasms is distinct from usual cases. However, no recommendation for the PCI procedure and risk management for the patient with classic myeloproliferative neoplasms. This case report will explore the angiographic features, technical aspects, and risk of no-reflow phenomenon in primary PCI for the patient with PV.

This case report presents a rare no-reflow phenomenon which occurred on the primary PCI procedure of multiple coronary arteries of the patient with PV. In this patient, PV may be related to the occurrence of the multiple coronary occlusion. Acute and multiple coronary occlusion led to STEMI. Therefore, the patient underwent primary PCI procedure. Catastrophic condition of no-reflow and ventricular fibrillation occurred during the procedure. We successfully manage this condition and the patient was successfully discharged at the 7th day after the procedure without recurrent thrombosis. The patient gave written consent for this publication and this case report also has been approved by the Ethics committee of the Soetomo General Hospital.

## 2 Patient concern

**Figure 1:**
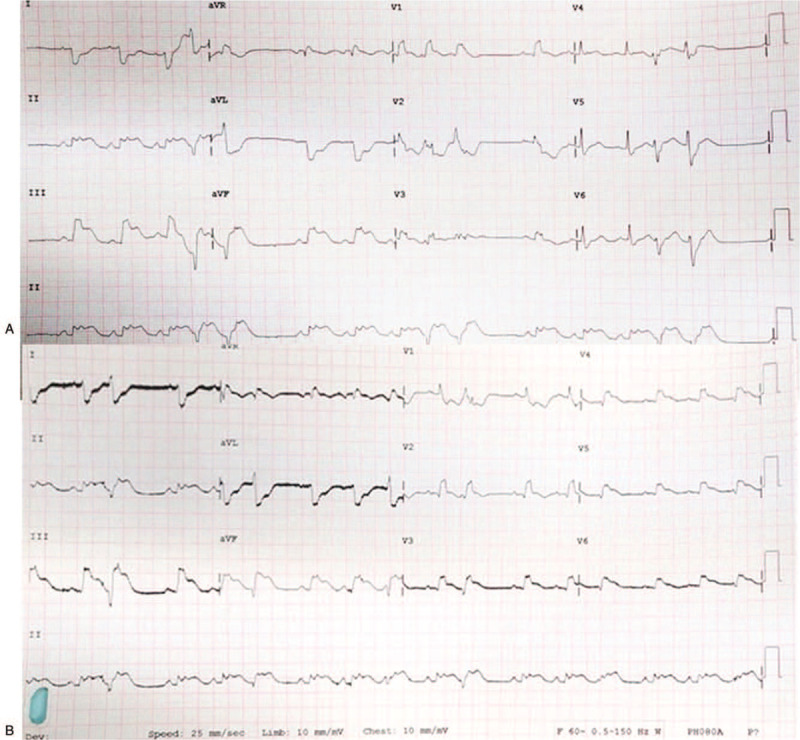
Admission ECG showing (A) ST-elevation in II, III, aVF, RBBB, and PVCs. (B) Right ECG showed ST-elevation in V4R–V6R. ECG = electrocardiography, PVC = primary ventricular contraction, RBBB = right bundle branch block.

A 46-year-old Indonesian woman was admitted to the tertiary care emergency department with the chief complaint of acute typical chest pain from 4 hours before admission. She felt dyspnea and diaphoretic. The patient's medical history showed a history of deep vein thrombosis at the left leg which was treated with low molecular weight heparin. Her physical examination showed blood pressure of 90/60 mmHg, pulse rate of 110 bpm and respiration rate of 28 times/min with low peripheral perfusion. On chest auscultation, Ronchi was found on the pulmonary base. Urgent bedside Electrocardiography (ECG) demonstrated sinus tachycardia 105 bpm, ST-elevation of 2 to 4 mm in II, III, aVF, V4R–V6R leads, complete right bundle branch block (RBBB), and couplet primary ventricular contraction (PVC) (Fig. 1). Laboratory assessment revealed Hs-Troponin T level of 496.4 pg/mL which indicates acute myocardial infarction.

Complete blood count test revealed erythrocytosis (hemoglobin = 21.2 g/dL; hematocrit = 66.1%), leukocytosis (white blood cell = 28.360/μL), and thrombocytosis (platelet = 1,342,000/μL). Genetic testing showed mutation on the JAK2 gene, which establishes the diagnosis of PV. Another laboratory results revealed metabolic acidosis, azotemia, and elevated liver enzymes indicating organ hypoperfusion due to cardiogenic shock. Aspirin 300 mg and clopidogrel 300 mg were administered orally. Oxygen supplementation and hemodynamic support using intravenous dopamine and furosemide were also given.

**Figure 2:**
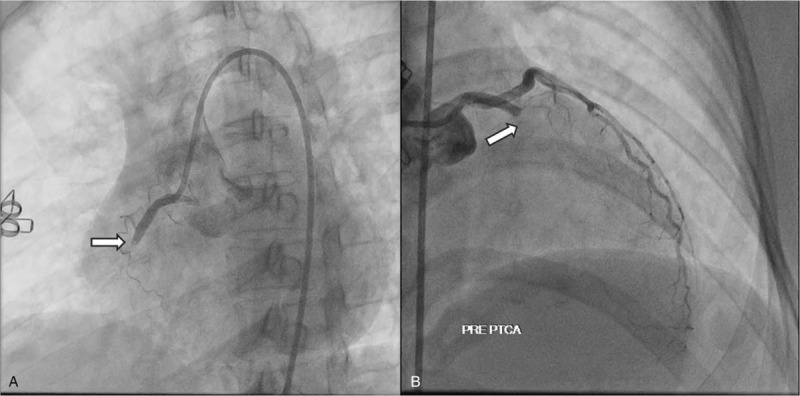
Acute simultaneous total occlusion at (A) proximal RCA, and (B) proximal LAD evaluated by diagnostic coronary angiography indicating large thrombus burden. LAD = left anterior descending, RCA = right coronary artery.

Diagnostic coronary angiography showed acute and simultaneous total occlusion in 2 major coronary arteries, which are total occlusion at the proximal part of the right coronary artery (RCA) and proximal part of left anterior descending (LAD) (Fig. 2). Based on the clinical sign, STEMI pattern on ECG, presence of simultaneous occlusion and hemodynamic instability, we then decided to perform multi-vessel primary PCI.

**Figure 3:**
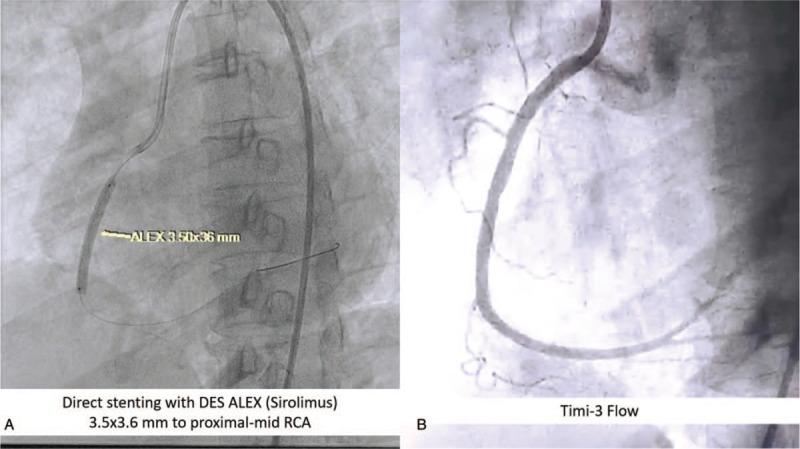
Percutaneous coronary intervention in the right coronary artery using a direct stenting method (A) result in TIMI-3 flow (B). TIMI = thrombolysis in myocardial infarction.

**Figure 4:**
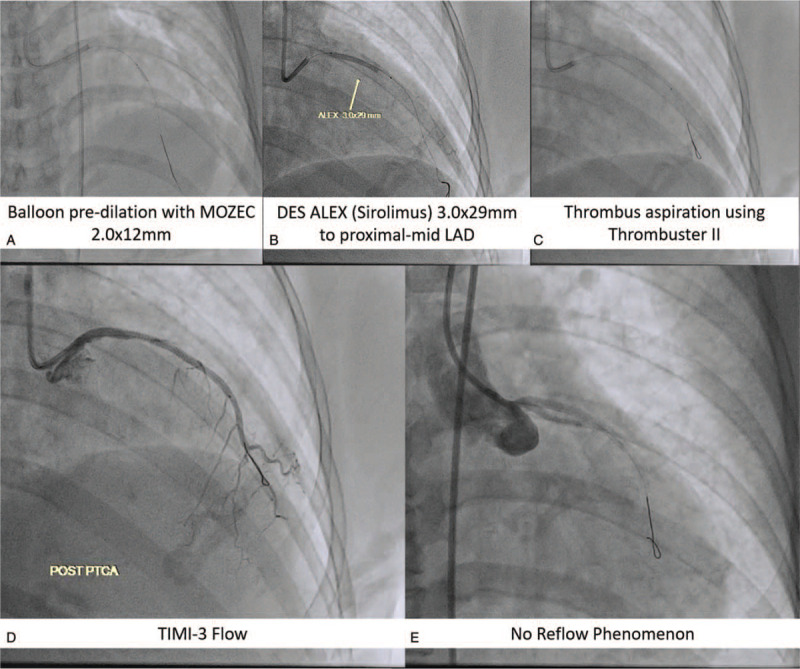
Percutaneous coronary intervention in the left anterior descending artery using conventional balloon pre-dilation technique, after failed insertion of aspiration catheter (A and B), resulted in TIMI-1 flow, followed by thrombus aspiration (C) result in transient TIMI-3 flow (D). Shortly the patient underwent ventricular fibrillation, due to no-reflow phenomenon (E). This complication might be due to distal embolization after balloon pre-dilation. TIMI = thrombolysis in myocardial infarction.

In the primary PCI, intravenous unfractionated heparin dose 6000 IU was administered. JR 4.0 7F guiding catheter was inserted and engaged in RCA ostium using 7F femoral sheath. NS run-through Hypercoat guidewire was successfully used to cross the lesion toward distal RCA. Thrombuster II 7F was inserted toward RCA for aspiration thrombectomy. We then deploy ALEX Drug-Eluting Stent (DES) 3.5 × 36 mm into proximal-mid RCA using direct stenting method (10 atm/10 s). Stenting resulted in thrombolysis in myocardial infarction (TIMI)-3 flow (Fig. 3). For LAD lesion, we used EBU 3.5 7F guiding catheter and inserted NS Run-through Hypercoat guidewire to cross the lesion toward distal LAD. Aspiration catheter was inserted to LAD, but we failed. This might due to stenotic lesion dominant at LAD. We then inserted MOZEC Balloon 2.0 × 12 mm to proximal LAD and dilated at 7 atm/8 s and 12 atm/4 s. However, balloon dilatation also failed to improve LAD flow. We then tried to insert ALEX stent (DES) 3.0 × 29 mm to cross the lesion and dilate at 8 atm/8 s. However, this method also failed to improve the flow (TIMI-1 flow). Lastly, thrombuster II 7F was inserted toward LAD and aspiration thrombectomy was done resulted in TIMI-3 flow (Fig. 4A–D).

**Figure 5:**
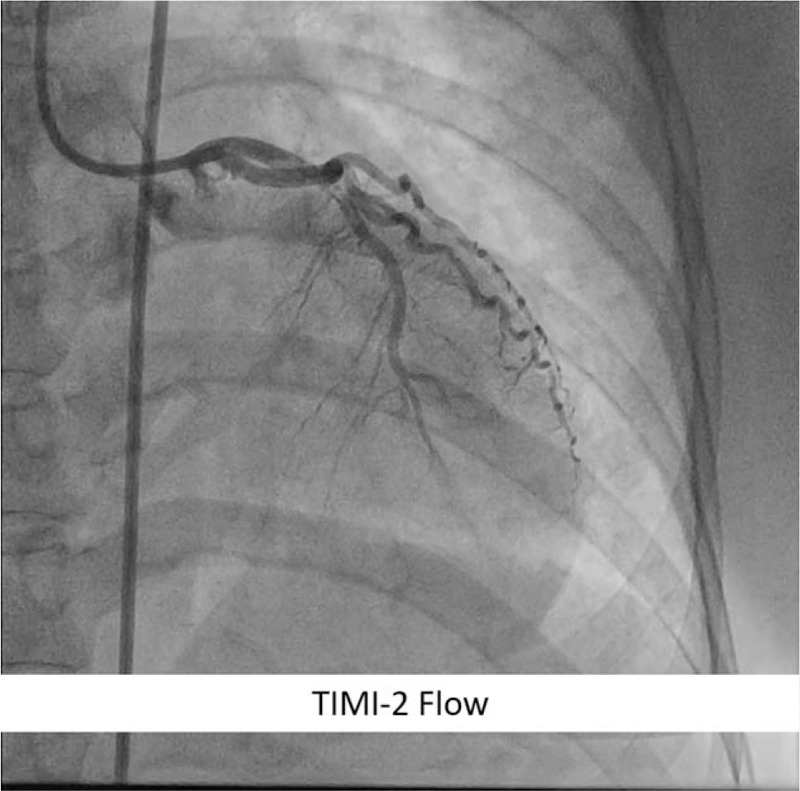
Final coronary angiogram at the left anterior descending artery after the management of the no-reflow phenomenon (resuscitation, repeated thrombus aspiration, and intracoronary thrombolysis) showed improved coronary flow (TIMI-2 flow). TIMI = thrombolysis in myocardial infarction.

**Figure 6:**
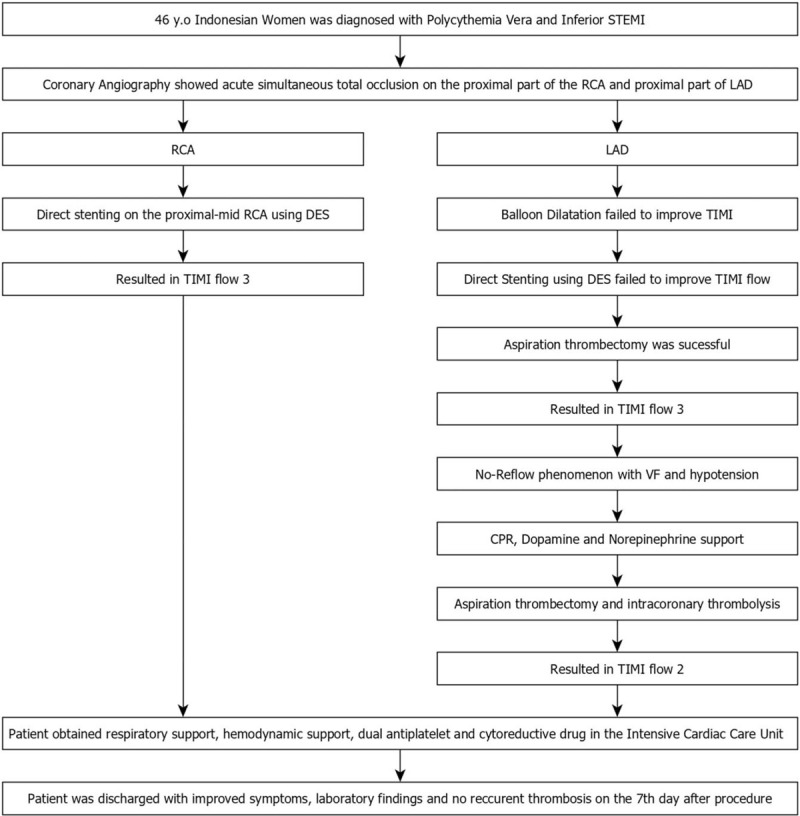
Workflow of the PV patient with multple coronary occlusion from the diagnosis, intervention, complication, treatment, and outcome. PV = polycythemia vera.

Shortly after the last angiogram, the patient rapidly became hemodynamically unstable with hypotension and ventricular fibrillation. Cardiopulmonary resuscitation was initiated through vigorous external cardiac massage, defibrillation, and endotracheal intubation. Intravenous dopamine and norepinephrine were administered. Evaluation angiogram revealed acute closure (no-reflow phenomenon) at LAD (Fig. 4E). Aspiration thrombectomy and low-dose intracoronary thrombolysis (at total 150,000 IU dose of streptokinase) were performed during cardiopulmonary resuscitation. After several attempts of resuscitation, the patient achieved the return of spontaneous circulation state and improved coronary flow in LAD (TIMI-2 flow) (Fig. 5). The procedure was stopped, and the patient was transferred to the intensive cardiac care unit and receives respiratory and hemodynamic support. Dual antiplatelet (aspirin 100 mg/d and clopidogrel 75 mg/d) and cytoreductive therapy using hydroxyurea 1500 mg/d were administered. The patient was stabilized with improved clinical and laboratory signs (hemoglobin: 18.5 g/dL, hematocrit: 59.6%, platelet: 725,000/μL), no sign of chest pain, and recurrent thrombosis. Patient was discharged at 7th day after procedure. The milestone of the case from the diagnosis to the outcome can be seen on the Fig. 6.

## 3 Discussion

PV is the example of classic MPNs which predominated with erythroid and megakaryocyte hyperplasia.^[4]^ According to the WHO diagnosis of PV is made from all 3 major or first major with 1 minor criteria (major criteria: 1) Hb >16.5 g/L for men and Hb >16 g/L for women or hematocrit of >49% for men and 48% for women; hypercellularity of bone marrow with accelerated growth of trilineage; and mutation of the JAK2 gene.^[1]^ Our patient had 2 major criteria and evidence of a coronary occlusion on this current presentation. Cardiovascular disease, especially myocardial infarction, is a fatal complication which happened in 9.4% of PV patient because PV is usually followed by thrombocytosis, which may increase the risk of thrombus occlusion in the coronary arteries.^[2]^

The main mechanism of polycythemia-related myocardial infarction is a complex combination of increased quantity and altered quality of blood cells. The quality alteration occurred are rheological abnormalities due to an increased red cell mass in PV; platelet function abnormalities; involvement of leukocytes and possibly of endothelial cells; and activation of the coagulation system which induce hypercoagulable state. All of the factors above may synergistically activate the platelet and induce thrombotic formation.^[5,6]^ The JAK2 gene mutations as a sign of PV diagnosis also shown to be correlated with the thrombosis event.^[7]^ All of these above were found in this patient and may responsible for the development of multiple coronary occlusion.

Acute simultaneous occlusion of 2 major coronary arteries in acute myocardial infarction is a rare condition. Clinically, it usually presents as cardiogenic shock and correlated with higher mortality and prolonged hospital stay with poor prognosis.^[8]^ Simultaneous occlusion may occur due to coronary vasospasm, cocaine abuse, hypercoagulability state, and coronary dissection. However, many cases also occurred without known underlying factors. In this patient, hypercoagulability due to PV and worsened hemodynamic state might be involved in the occurrence of simultaneous coronary occlusion.^[8,9]^ Large thrombus burden in this patient also may contribute to the occlusion of 2 major coronary arteries.

Primary PCI is the most recommended treatment of simultaneous coronary occlusion case. While primary PCI in the infarct-related coronary artery is highly recommended, analysis of the lesion type should be done to determine the best revascularization procedure.^[3]^ In this case, inferior and right ventricle STEMI pattern in ECG indicates occlusion of the RCA. RBBB pattern in this patient also could indicate an occlusion in the proximal of the LAD septal branch, which vascularized the right bundle branch.^[10]^ According to the latest STEMI algorithm for multi-vessel diseases, multi-vessel primary PCI is the most suitable strategy in the presences of multiple infarct-related arteries with hemodynamic instability.^[11]^

Several cases reported that STEMI patient with PV usually followed by worsening of acute coronary syndromes. However, no uniform strategy was used in the PCI procedure of the patient with PV. Previous case reports have shown that the management of the patient with PV and STEMI may vary depending on the existence and location of the thrombus. The outcome also varies from death, acute stent thrombosis to good.^[12–15]^ Venegoni and Schroth,^[12]^ reported that in the patient with PV, occlusion by thrombus in the proximal LAD, which managed through primary PCI with intracoronary urokinase and ballon angioplasty resulted in death as an outcome. Inami et al,^[15]^ reported that similar thrombus occlusion at proximal LAD which managed through primary PCI with Bare Metal Stent stenting resulted in acute stent thrombosis as an outcome. Interestingly, the good outcome was observed on the thrombolysis treatment on a patient with extensive anterior STEMI and PV,^[14]^ also on the conservative treatment of a patient with anterior STEMI and PV.^[13]^

An international guideline has provided the technical guidelines for the primary PCI in the patient with STEMI.^[3]^ However, the presences of large thrombus burden in the patient with PV may be associated with the adverse procedural complication. Hence, identifying the most suitable STEMI management in a patient with PV require a good judgment on the clinical state, pathophysiology, hemodynamic, resources, and operator's experience.^[16]^ In this case, we used thrombus aspiration, direct stenting, and also intracoronary thrombolysis. Thrombus aspiration was used primarily in the aspiration catheter which usually contains 2 lumina, first lumen for the guidewire and second (larger lumen) for aspiration. This catheter is connected to a negative pressure device at the proximal, and then the operator can advance the catheter to distal through a coronary lesion to evacuate thrombus.^[17]^ This technique was able to reduce thrombus load, minimize the need for balloon pre-dilation, facilitate direct stenting, prevent distal embolization, and improve myocardial reperfusion. However, recent large studies have revealed that this technique is not associated with the decrease of major adverse cardiac events and even it is associated with higher stroke risk.^[18,19]^ Hence, thrombus aspiration is not recommended routinely.^[3]^ However, some observational studies have suggested the benefit of thrombus aspiration in the large thrombus burden.^[20]^ This case was an example of the beneficial effect of thrombus aspiration in a large thrombus burden case.

Stenting technique selection is also an important consideration in the PCI procedure. Previous studies conclude that direct stenting has advantages through a decrease in vessel injury, distal embolization, and no-reflow in the thrombus containing lesion.^[21]^ In this patient, PV has increased thrombus burden which causes thrombus occlusion. Hence, we proposed a direct stenting method as the first choice in the patient with PV combined with intracoronary thrombolysis to resolve the thrombosis burden. Intracoronary thrombolysis has been used as therapy in acute thrombotic occlusion where angioplasty is not technically feasible or thrombus-related acute closure.^[22]^ Recent studies also showed a potential effect of the intracoronary thrombolysis for the patient after failed thrombectomy or in combination with thrombectomy.^[23,24]^ In this patient, we successfully manage the no-reflow phenomenon through low-dose intracoronary thrombolysis. Similar to this case, the previous study shows the beneficial effect of low-dose intracoronary thrombolysis in STEMI patients with a large thrombus burden and significant residual thrombus after manual aspiration.^[25]^

After the PCI procedure, risk-adapted therapy should be used to prevent recurrent thrombotic complications. In high-risk patient, including PV, hydroxyurea should be given to minimize the thrombosis risk through inhibition of deoxyribonucleic acid synthesis in bone marrow tissue. Low dose aspirin is recommended to minimize thrombosis risk in the PV patient.^[4]^ Dual antiplatelet therapy is also recommended to prevent stent thrombosis and adverse effects after PCI.^[26]^ In this patient, cytoreductive and dual platelet therapy was used and resulted in no thrombosis event and quick recovery after PCI procedure, suggesting their benefit for the patient with PV after undergoes PCI.

## 4 Conclusion

Pathophysiology and awareness of the large thrombus burden are the keys in primary PCI technique selection in the PV patients with STEMI. Aspiration thrombectomy and direct stenting (instead of conventional balloon pre-dilation) are proposed to be the first-choice technique in primary PCI in a patient with PV. Interventionist should be aware of the no-reflow phenomenon risk and can consider intracoronary thrombectomy and thrombolysis to overcome this complication.

## Acknowledgments

The authors are grateful to the patient and colleagues from Soetomo General Hospital and Siloam Hospital who were involved in this work.

## Author contributions

**Conceptualization:** Yudi Her Oktaviono, Suryo Ardi Hutomo.

**Data curation:** Yudi Her Oktaviono.

**Formal analysis:** Suryo Ardi Hutomo.

**Funding acquisition:** Yudi Her Oktaviono.

**Investigation:** Suryo Ardi Hutomo, Makhyan Jibril Al-Farabi.

**Project administration:** Suryo Ardi Hutomo, Makhyan Jibril Al-Farabi.

**Resources:** Suryo Ardi Hutomo.

**Supervision:** Yudi Her Oktaviono.

**Validation:** Yudi Her Oktaviono, Makhyan Jibril Al-Farabi.

**Visualization:** Makhyan Jibril Al-Farabi.

**Writing – original draft:** Suryo Ardi Hutomo, Makhyan Jibril Al-Farabi.

**Writing – review & editing:** Yudi Her Oktaviono, Makhyan Jibril Al-Farabi.
